# Identification of *Colletotrichum* Species Associated with Blueberry Anthracnose in Sichuan, China

**DOI:** 10.3390/pathogens9090718

**Published:** 2020-08-31

**Authors:** Xuan Liu, Xiaojuan Zheng, Muhammad Ibrahim Khaskheli, Xiaofang Sun, Xiaoli Chang, Guoshu Gong

**Affiliations:** 1 Plant Protection Department, College of Agronomy, Sichuan Agricultural University, Chengdu, Sichuan 611130, China; lx665461@163.com (X.L.); zhengxiaojuan0805@163.com (X.Z.); sunxiaofang207@163.com (X.S.); xl_chang14042@sicau.edu.cn (X.C.); 2 Department of Plant Protection, Sindh Agriculture University, Tandojam 70060, Pakistan; mikhaskheli@sau.edu.pk

**Keywords:** blueberry, anthracnose, *Colletotrichum* spp., multilocus phylogeny, pathogenicity

## Abstract

Anthracnose caused by *Colletotrichum* spp. is an important disease of blueberries and results in large economic losses for blueberry growers. Samples of anthracnose were collected from six main blueberry cultivation areas in Sichuan Province. In total, 74 *Colletotrichum* isolates were obtained through a single-spore purification method and identified to the species through morphological characteristics and phylogenetic analyses based on partial DNA sequences of glyceraldehyde-3-phosphate dehydrogenase (*GAPDH*), internal transcribed spacer (*ITS*) regions, and the β-tubulin (*TUB2*), actin (*ACT*) and calmodulin (*CAL*) genes. Among all species, *Colletotrichum fructicola* was the most dominant species, with an isolation percentage of up to 66.2% in Sichuan, followed by *Colletotrichum siamense* (17.6%), *C. kahawae* (5.4%), *C. karstii* (5.4%), *C. nymphaeae* (2.7%) and *C. sichuaninese* (2.7%). Pathogenicity tests showed all species were able to cause typical anthracnose symptoms on blueberry leaves and stems. *Colletotrichum fructicola* was the predominant species with strong aggressiveness. Moreover, *C. fructicola*, *C. kahawae, C. sichuaninese* and *C. nymphaeae* are first reported here to cause blueberry anthracnose. This study provides a comprehensive reference for the association of different *Colletotrichum* spp., which may support the sustainable management of blueberry anthracnose.

## 1. Introduction

Blueberry (*Vaccinium corymbosum*) belongs to the family Ericaceae, subfamily Vaccinoideae [1]. Blueberries are rich in antioxidants, anthocyanins, tannins and folic acid, which help prevent and delay aging [2]. Therefore, the market demand is expanding, global blueberry production is increasing, and the price of blueberries continues to increase [3]. Because of their high nutrient and business prospects, blueberries have been widely cultivated worldwide [4,5]. The increased cultivation of blueberries drastically promotes severe infectious diseases that cause significant economic losses to the blueberry industry. Among these diseases, blueberry anthracnose has been considered one of the most serious diseases causing economic damage in North America [6], Japan [7], Spain [8], South Korea [9], China [10], and other countries worldwide. Due to the high humidity conditions in Sichuan Province, the incidence of anthracnose in major blueberry regions usually reaches 30%, and the disease worsens with tree age, resulting in severe deciduous leaves or dead trees, with yields decreasing by 10~15% in some severe regions.

Previous reports have shown that blueberry anthracnose is mainly caused by *C. acutatum* J.H. Simmonds [8,9,11] and *C. gloeosporioides* Penz., as based on morphological identification [9,12,13]. In 2015, *C. karstii* was also recognized as a pathogen causing blueberry anthracnose in Brazil [14]. In China, this disease was first reported in 2012 in Liaoning Province and was caused by *C. gloeosporioides* and *C. acutatum* [15,16]. Since then, the identification of blueberry anthracnose has rarely been reported.

Three classification systems mainly based on morphological characteristics have been used to identify *Colletotrichum* species [17]. The first traditional classification system was presented by Saccardo in 1882. Later, in 1957, the Von Arx classification system was introduced, which established the first taxonomic system based on the morphology of the *Colletotrichum* genus. In 1980, Sutton improved Von Arx’s system and advocated different criteria for species identification with respect to morphological characteristics, host range and pathogenicity. However, it is still very difficult to distinguish the majority of similar species merely on the basis of morphological characteristics [18,19,20,21]. With the molecular characterization of the pathogen and the development of DNA sequencing over past decades, a combination of morphological characteristics and multilocus phylogeny has recently been used to identify *Colletotrichum* species. To date, many anthracnose diseases have been diagnosed in various crops, such as apple [22], grapevine [23], pepper [24], jute [25], persimmon [26] and citrus [27].

Sichuan Province is the main blueberry cultivation area in China and marked blueberry anthracnose has occurred in recent years. However, the pathogens causing this disease, have not yet been reported. Therefore, the objective of the current study was to clarify the composition of *Colletotrichum* species on blueberries in China using a combination of morphological characteristics and multiple locus phylogenetic analysis. Our study reveals the associations of *Colletotrichum* species with blueberries and may help growers to sustainably manage blueberry anthracnose.

## 2. Results

### 2.1. Symptomatology and Fungal Isolation

**Figure 1:**
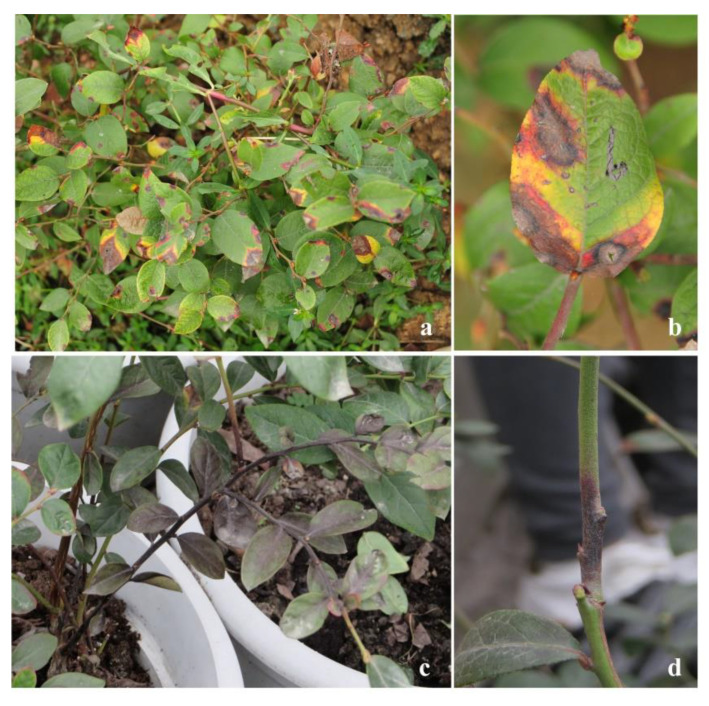
Symptoms of anthracnose on blueberries under field conditions. (**a**) Spots on blueberry leaves; (**b**) a close-up image of diseased leaves; (**c**) stems blackened from the base; (**d**) stems blackened from the middle.

Based on field observations, anthracnose symptoms on leaves and stems were recorded. The disease started at the leaf margin, evidenced by a red halo at the junction of diseased and healthy portions, and the center of the diseased spot was dark gray and irregularly expanded. Then, leaf blight developed, and a yellow color was observed (Figure 1a,b). The diseased stems showed irregular black spots that started from the base or middle, and then the whole branches withered (Figure 1c,d). From 2016 to 2018, 74 isolates of *Colletotrichum* were isolated from 85 samples (Table 1). Among 48 isolates obtaind from leaves, 30 isolates were identified as *C. fructicola*, 10 as *C. siamense*, and the remaining 8 as other *Colletotrichum* species. In total, 26 isolates were isolated from stems; 19 isolates were identified as *C. fructicola*, and the remaining 7 isolates belonged to other *Colletotrichum* species. Other fungi, such as *Alternaria* and *Pestalotiopsis*, were also isolated, but they were not considered in this study.

### 2.2. Morphological Characterization

**Figure 2:**
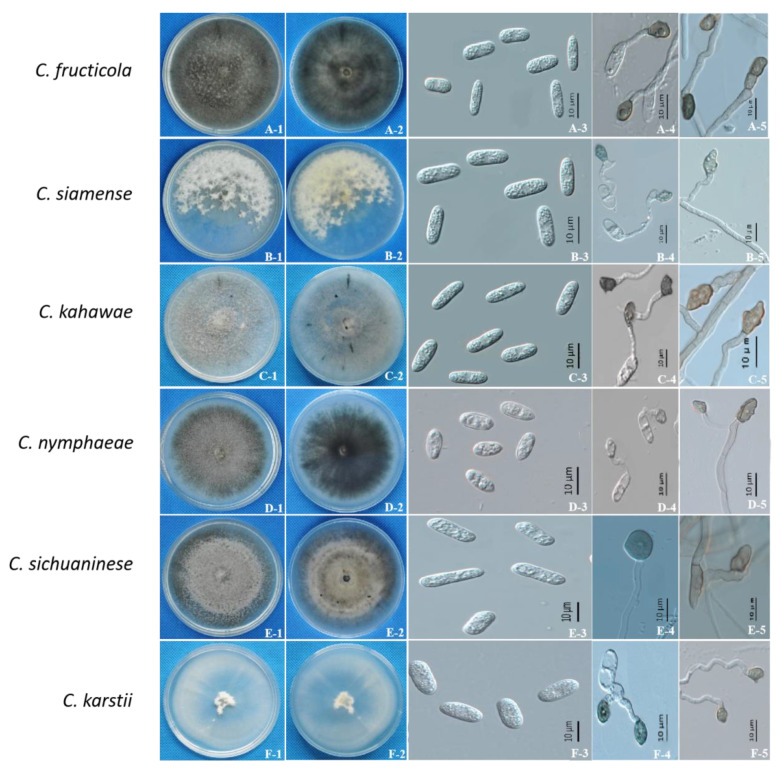
Illustration of the morphological and cultural characteristics of *Colletotrichum* spp. associated with blueberry anthracnose in Sichuan. Each row from left to right: views of the surface and the bottom of potato dextrose agar (PDA) plate of representative isolates, conidia, conidial appressoria and mycelial appressoria.

After a comprehensive morphological analysis, all isolates (*n* = 74) were classified into six morphological groups (Figure 2). The morphological characteristics of six *Colletotrichum* spp. (*C. fructicola*, *C. siamense*, *C. kahawae*, *C. karstii*, *C. nymphaeae* and *C. sichuaninese*) were obviously different, and are provided below in detail.

**Figure 3:**
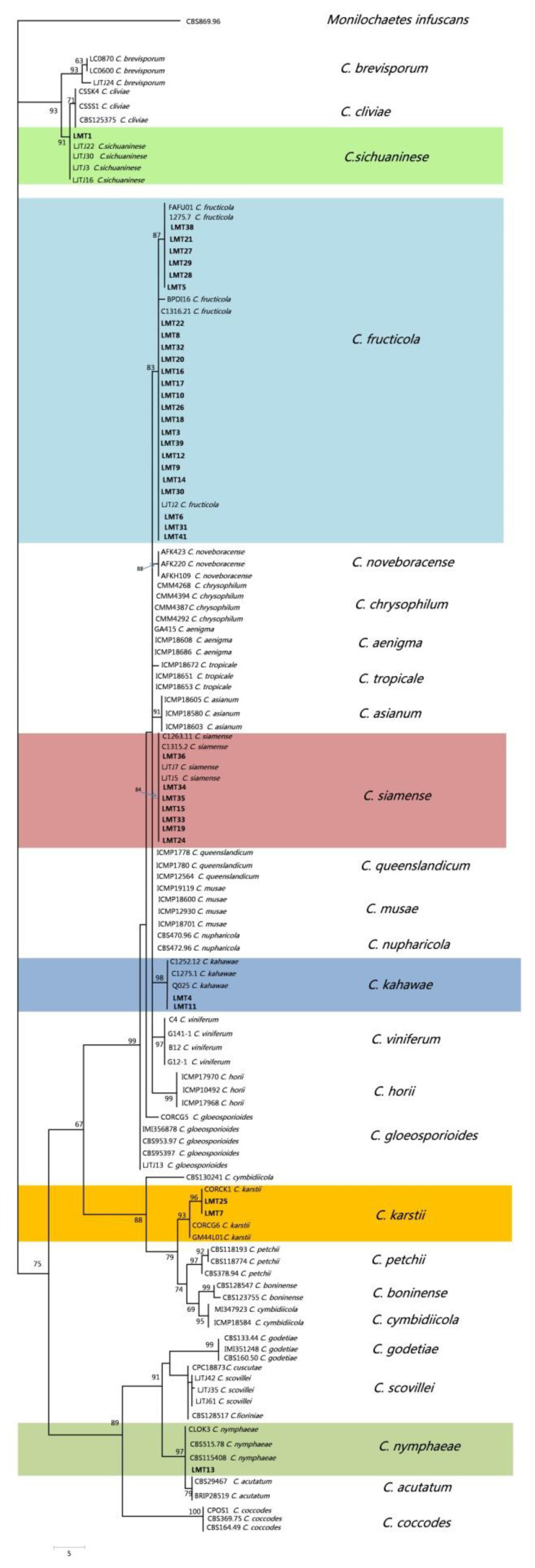
Phylogenetic tree generated from maximum parsimony analysis based on *GAPDH*, *ITS*, *TUB2*, *ACT* and *CAL* gene sequences. Parsimony bootstrap values of more than 50% are shown at the nodes. Isolates from this study are shown in bold. The tree is rooted with *Monilochaetes infuscans*. Detailed information is provided in Table S1.

**Figure 4:**
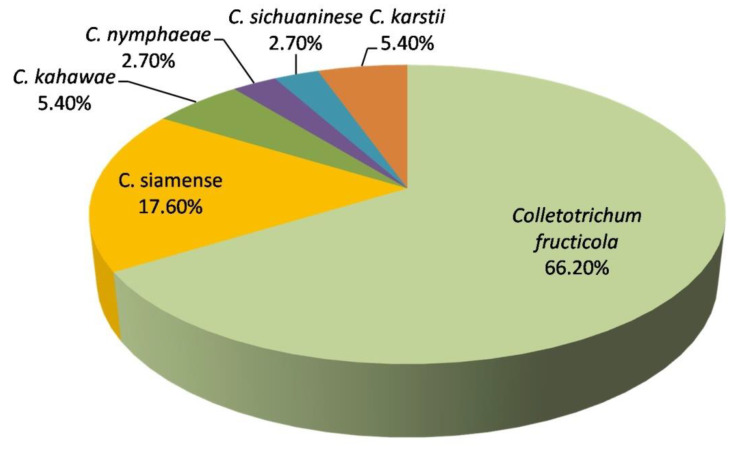
The proportion of *Colletotrichum* species associated with blueberry anthracnose in Sichuan.

*Colletotrichum fructicola*, *C. kahawae*, *C. sichuaninese* and *C. nymphaeae* are reported as novel pathogens of blueberries. In this study, *C. fructicola* developed a black-colored colony with a thin aerial hyphal growth, and developed gray, orange-red uredinia after approximately 7 days of incubation (Figure 2A-1). However, the colonies of the other three species were gray and fluffy aerial hyphae after incubation (Figure 2). *Colletotrichum kahawae* had the slowest average mycelial growth rate (3.4 ± 0.09 mm/d) (Table 2). The conidia of *C. nymphaeae* were fusiform (Figure 2D-3), but *C. fructicola, C. kahawae* and *C. sichuaninese* conidia were cylindrical in shape (Figure 2A-3,C-3,E-3). The growth rate (6.2 ± 0.08 mm/day) of *C. nymphaeae* was higher than *C. siamense, C. kahawae* and *C. sichuaninese* (Table 2). The spore appressorium of *C. nymphaeae* was gray and round (Figure 2D-4), but that of *C. fructicola, C. kahawae* and *C. sichuaninese* was brown to dark black (Figure 2A-4,C-4,E-4).

**Figure 5:**
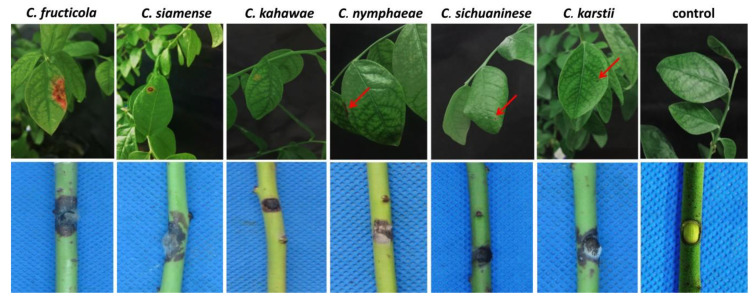
Symptoms of blueberry leaves and detached stems inoculated with different *Colletotrichum* species. Note: Representative isolates of *C. fructicola*, *C. siamense, C. kahawae, C. nymphaeae, C. sichuaninese* and *C. karstii* are LMT12, LMT19, LMT4, LMT13, LMT1 and LMT25, respectively; the control was inoculated with sterile water or only PDA plugs. The red arrows indicate the site of the lesions.

*C. siamense* and *C. karstii* have been reported to cause blueberry anthracnose in other areas [14,28]. The colonies of *C. siamense* were white and developed sparse and white aerial hyphae with orange-red spores after approximately 10 days of incubation (Figure 2B-1). The mycelial appressoria were irregularly shaped and brown, with a deep, marginal, lobed edge (Figure 2B-5). *C. karstii* developed white colonies with thin aerial hyphae after approximately 6 days of incubation (Figure 2F-1). The spore appressorium and mycelial appressoria were round (Figure 2F-4,F-5), but the spore appressorium of *C. siamense* was irregular (Figure 2B-4).

### 2.3. Molecular Characterization and Phylogeny of Isolates

A maximum-parsimony tree and maximum likelihood tree were constructed based on the GAPDH gene region using 74 isolates obtained in this study (Figures S1 and S2). Based on the 260 conserved characteristics used in the phylogenetic analysis, the tree contained six primary clades. After a comprehensive analysis of morphological characteristics and the *GAPDH* gene tree, all isolates were divided into the 6 different groups as explained above, and 37 representative isolates were further selected from different clades for molecular identification. A multilocus phylogenetic tree, based on 2335 characters consisting of *ITS*, *TUB2*, *ACT*, *CAL* and *GAPDH*, was constructed using 121 isolates of *Colletotrichum* (37 from this study) and *Monilochaetes infuscans* (CBS869.96) as the outgroup; the other 84 isolates of *Colletotrichum* species reported on different hosts were obtained from GenBank (Table S1).

Both maximum parsimony (MP) and maximum likelihood (ML) analyses revealed that the 37 representative isolates selected in this study clustered with *C. fructicola*, *C. siamense*, *C. kahawae*, *C. karstii*, *C. nymphaeae* and *C. sichuaninese*. Phylogenetic analyses performed using the maximum parsimony and maximum likelihood method produced similar topologies; only the combined dataset based on maximum parsimony is shown in Figure 3 (CI = 0.615, RI = 0.927, RC= 0.570), and the maximum likelihood tree in Figure S3. We found that the isolates from Group 1 clustered with *C. fructicola*, including the majority of isolates in this study; those from Group 2 clustered with *C. siamens*e, and those from Group 3 clustered with *C. kahawae*. The isolates composed a highly supported clade (98% bootstrap support) with C1275.1 in Arabian coffee from Angola, C1252.12 in Kunzea ericoides from New Zealand and Q025 in blackberry from Colombia. The isolates from Group 4 clustered within *C. nymphaeae*, those from Group 5 clustered with *C. sichuaninese*, and those from Group 6 clustered with *C. karstii* (CORCG6, CORCK1, GM44L01), with strong bootstrap support of 96%.

According to morphological characteristics and molecular analysis, the identification of the 74 isolates showed that *C. fructicola* was the most dominant species (66.2%), followed by *C. siamens*e (17.6%), *C. kahawae* (5.4%), *C. karstii* (5.4%), *C. nymphaeae* (2.7%) and *C. sichuaninese* (2.7%) (Figure 4). The sequences of all the representative isolates in the current study have been deposited in the NCBI database GenBank (Table S1).

### 2.4. Pathogenicity

The representative isolates (*n* = 37) of the six different species isolated, that grew on PDA medium at 28 °C for 5 days, were inoculated onto one-year-old healthy potted blueberry plants cv. O’Neal. The plants displayed disease symptoms at 7 days of inoculation, showing that all these isolates were able to infect blueberry leaves and detached stems; however, inoculation of the attached blueberry stems did not result in any symptoms (Figure 5). The typical symptoms produced by artificial inoculation conformed with the original symptoms under natural conditions, and Koch’s postulate was verified by identifying reisolated strains based on morphological characteristics and ITS sequences. Moreover, different species induced variable pathogenic effects on blueberry tissues and showed distinct aggressiveness. However, *C. fructicola* was the species with the highest aggressiveness on attached leaves and detached stems. At 3 days after inoculation, symptoms began to appear on leaves and stems; 5 days after inoculation, the symptoms on the leaves were significantly expanded, and abundant mycelia and orange spores appeared on the detached stems. In contrast, *C. nymphaeae* and *C. karstii* were weakly aggressiveness to leaves, and at 7 days after inoculation, the symptoms on leaves did not markedly spread. *C. sichuaninese*, *C. kahawae* and *C. nymphaeae* were weakly pathogenic on detached stems, no orange spores were seen on the stems, and the symptoms did not markedly spread.

We also evaluated the pathogenicity of six *Colletotrichum* species via the diameter of lesions. Different species showed differences in pathogenicity (Table 3); the lesion diameters of leaves were 5.8–14.5 mm, and the lesion diameters of stems ranged from 1.4 mm to 15.2 mm. In terms of each species, *C. fructicola* has strong aggressiveness, and at 5 days after inoculation, both leaves and stems had significantly larger lesions (14.2 ± 0.35 mm, 15.0 ± 0.20 mm). Conversely, *C. sichuaninese* showed weak pathogenicity, with a small range of lesions on stems and leaves after inoculation (6.0 ± 0.22 mm, 1.6 ± 0.10 mm).

## 3. Discussion

In our study, 74 isolates were obtained from 85 blueberry anthracnose samples (leaves and stems), mainly collected from major blueberry cultivation areas in Sichuan Province, China. Six species of *Colletotrichum* associated with blueberry anthracnose were characterized using a phylogenetic approach. *Colletotrichum fructicola, C. siamense* and *C. kahawae* belong to the *C. gloeosporioides* species complex, and *C. nymphaeae* and *C. karstii* belong to the *C. acutatum* species complex and the *C. boninense* species complex, respectively. *C. sichuaninese* belongs to the *C. orchidarium* species complex. *Colletotrichum fructicola, C. kahawae*, *C. sichuaninese* and *C. nymphaeae* were first recorded in this study to cause blueberry anthracnose.

Although *C. gloeosporioides* and *C. acutatum* have previously been identified as the main causal agents of blueberry anthracnose in different countries worldwide [8,9,15,16,29,30], we found that none of the isolates from blueberry in the current study belong to *C. gloeosporioides* species sensu stricto and the *C. acutatum* species sensu stricto. Since the early identifications were mainly based on morphological characterization and *ITS* sequence, which have been shown to be unable to distinguish among certain species of *Colletotrichum*, we speculate that the early reported species were probably species complexes of *C. gloeosporioides* and *C. acutatum*, respectively. Our results appear to be quite different from previous studies, with most isolates mainly belonging to the *C. gloeosporioides* species complex and the *C. acutatum* species complex, and accounting for 92% of all the isolates in this study. From the perspective of the species complex, the results are basically consistent with previous studies. Six species were found through the use of multiple representative genes (*GAPDH*, *BAT*, *CAL*, *ACT* and *ITS*) in phylogenetic analyses in this study, indicating the very rich diversity of the *Colletotrichum* species in blueberries in Sichuan. It is speculated that the diversity of *Colletotrichum* may be detected through polygenic phylogenetic analysis in other regions worldwide, for example, *C. karstii* was first found on blueberry anthracnose in Brazil [14].

According to the result of our study, *C. fructicola* was the dominant and highly aggressive pathogen causing the disease. *Colletotrichum fructicola* is a nonhost-specific pathogen that has been reported on certain hosts, such as apple [22], *Citrus* spp. [31,32], *Pyrus* spp. [33], *Camellia sinensis* [34], *Capsicum* sp. [24,35], *Mangifera indi*ca [36,37] and *Malus* sp. [38]. Although *C. sichuaninese* was first observed on pepper in Sichuan Province [24], to the best of our knowledge, this is the first recorded species to cause anthracnose disease in blueberries worldwide. As the wide host range of *C. sichuaninese* may pose a serious threat to the blueberry industry, further work will focus on the diversity of the *Colletotrichum* species, the nonhost specificity of most species and the characteristics of the newly recorded species associated with blueberries in Sichuan.

In conclusion, this study is the first detailed report of blueberry anthracnose in China and provides a basis for disease management. Nonetheless, the pathogenic mechanisms of different *Colletotrichum* spp. in blueberry need to be further studied.

## 4. Materials and Methods

### 4.1. Sample Collection and Isolation

From 2016 to 2018, a total of 85 samples of blueberry leaves (*n* = 48) and stems (*n* = 37) showing typical symptoms of anthracnose on three blueberry cultivars were collected from six blueberry growing areas in Sichuan Province, China (Table 1). Some samples came from the same blueberry growing areas, but from different orchards; approximately 20 samples were collected in a 1 hectare orchard. The samples were stored in sterile polythene bags and brought to the laboratory for stereomicroscope (OLYMPUS SZX16, Tokyo, Japan) examination. Then, lesion margin pieces (5 × 5 mm) were cut and sterilized with sodium hypochlorite for 30 s, and mercuric chloride for 25 s, rinsed three times with distilled sterile water, cultured on potato dextrose agar (PDA), and inoculated in a chamber at 28 ± 1 °C with a 12 h photoperiod for 3 days [39]. Single-spore isolates were obtained from cultures on the PDA plates according to Gong et al. [40]. A single spore was inoculated with sterilized acupuncture needles onto a water agar block, and the block was moved to new plates that were incubated at 28 ± 1 °C for 3 days. Later, the hyphal block was transferred to PDA plates and incubated at 28 °C for 5 days. The pure cultures were obtained and stored on PDA slants at 4 °C.

### 4.2. Morphological Characterization

After culturing on PDA at 28 °C for 5 days, the morphological characteristics of all isolates, including colony, conidia, conidial appressoria and mycelial appressoria, were initially observed. The colony diameter of each isolate from 1 day to 7 days after inoculation was assessed using the cross measurement method [41]. The size of approximately 30 conidia was measured and recorded. Conidial appressoria were induced at 27 °C for 24 h [42], and 30 appressorial sizes from each isolate were randomly measured. Mycelial appressoria were observed by the slide culture technique [43], and the development of mycelia was examined after 7 days of incubation at 28 °C. All developmental stages were observed with a Carl Zeiss Microscope (GmbH37081, Gottingen, Germany). The data on the radial colony growth rate, conidial size and conidial appressoria size were statistically analyzed for variance at the *p* = 0.05 level (LSD) using SPSS v21.0 software.

### 4.3. Molecular Characterization

The aerial mycelia of all isolates grown on potato dextrose agar (PDA) for 5 days at 25 °C in the dark were collected for DNA extraction according to the protocol by Than et al. [44]. First, the glyceraldehyde-3-phosphate dehydrogenase (*GAPDH*) gene was amplified to select representative isolates [24]. The nuclear rDNA *ITS* region and the β-tubulin (*TUB2*), partial actin (*ACT*) and calmodulin (*CAL*) genes were amplified for each representative isolate using the primer pairs ITS1/ITS4 [45], Bt2a/Bt2b [46], ACT512F/ACT783R [47] and CL1/CL2A [48], respectively. PCR was performed by following the protocol described by Prihastuti et al. [49]. PCR was conducted on 30 µl: 0.5 µL upstream primers (10 mol/L), 0.5 µL downstream primers (10 mol/L), 1 µL DNA templates (30–50 ng/µL), 15 µL 2 × PCR Mastermix and 13 µL ddH_2_O. The PCR-amplified product was examined by 1% agarose gel electrophoresis and sequenced by Shanghai Biological Engineering Co. Ltd., P. R. China.

### 4.4. Phylogenetic Analysis

Phylogenetic analysis was conducted by the method described by Liu et al. [24]. The *GAPDH* genes of the isolates (*n* = 74) were blasted to compare the sequence identity with those in the NCBI database and analyzed using Clustal X 1.81. Next, the maximum parsimony and maximum likelihood analyses were used to construct a phylogenetic tree in MEGA 6. Representative isolates (*n* = 37) were selected according to the morphological characterization and *GAPDH* gene tree and were combined with their high-similarity isolates retrieved in the NCBI database and type specimens [19,20,21,42,50] (Table S1) to build polygenic trees (*ITS*, *TUB2*, *ACT*, *CAL* and *GAPDH*) by the maximum parsimony and maximum likelihood analyses. The consistency index (CI), retention index (RI) and rescaled consistency index (RC) were also calculated.

### 4.5. Pathogenicity Tests

The pathogenicity of representative isolates (*n* = 37) for the six species was tested on one-year-old healthy potted blueberry plants (cv. O’Neal). The plants were surface sterilized with 75% alcohol and then rinsed three times with sterile distilled water before being used for the following inoculations. All isolates were grown on PDA medium at 28 °C for 5 days, and then spore suspensions at a concentration of 1 × 10^5^ conidia/mL or 5 mm diameter mycelial plugs were selected for inoculation. Sterile water or only PDA plugs were used as controls. For the inoculation of leaves in vivo, the leaves of potted plants were sprayed with 1 × 10^5^ conidia/mL spore suspensions using a handheld sprayer. For inoculation of stems in vitro, the stems were cut into 8 cm lengths, and 5 mm diameter mycelial plugs were applied to a wound of stems made using a sterile cork borer. The inoculated stems were then placed on a disk and covered with plastic wrap [51]. For the inoculation of stems in vivo, wounds were generated with a sterilized pointed needle in the middle of the potted plant stem and then inoculated with 5 mm diameter mycelial plugs. Five healthy leaves and three stems were inoculated on different plants as one replicate, and three replicates were used for each treatment and control. All treated samples were incubated in a humid atmosphere for 25 °C in a greenhouse until disease symptoms appeared. All experiments were repeated twice. Symptoms were recorded every day, and the diameters of the lesions were measured at 5 days after inoculation. The data were analyzed with SPSS Statistics 21.0 by one-way ANOVA.

The inoculated leaves and stems were reisolated to confirm the pathogen identity based on morphological characteristic and ITS sequence analyses.

## Figures and Tables

**Table 1:** List of different isolates with their geographical information isolated from blueberry anthracnose in Sichuan Province.

Isolate No.	Parts	Cultivars	Location	Latitude and Longitude	Species
LMT1	Leaf	Sharpblue	Daguang, Dujiangyan	N 30°51′4″ E 103°34′44″	*C. sichuaninese*
LMT2	Leaf	Sharpblue	Daguang, Dujiangyan	N 30°51′4″ E 103°34′44″	*C. fructicola*
LMT3	Stem	Misty	Tuqiao, Jintang	N 30°31′17″ E 104°50′5″	*C. fructicola*
LMT4	Stem	Misty	Tuqiao, Jintang	N 30°31′17″ E 104°50′5″	*C. kahawae*
LMT5	Leaf	Misty	Taiping, Shuangliu	N 30°25′53″ E 104°11′54″	*C. fructicola*
LMT6	Leaf	O’Neal	Taiping, Shuangliu	N 30°25′53″ E 104°11′54″	*C. fructicola*
LMT7	Leaf	Sharpblue	Daguang, Dujiangyan	N 30°51′4″ E 103°34′44″	*C. karstii*
LMT8	Leaf	Sharpblue	Daguang, Dujiangyan	N 30°51′4″ E 103°34′44″	*C. fructicola*
LMT9	Stem	O’Neal	Caichang, Dayi	N 30°30′8″ E 103°41′57″	*C. fructicola*
LMT10	Stem	O’Neal	Caichang, Dayi	N 30°30′8″ E 103°41′57″	*C. fructicola*
LMT11	Leaf	Misty	Tuqiao, Jintang	N 30°31′17″ E 104°50′5″	*C. kahawae*
LMT12	Leaf	Misty	Daguang, Dujiangyan	N 30°51′4″ E 103°34′44″	*C. fructicola*
LMT13	Leaf	Sharpblue	Caichang, Dayi	N 30°30′8″ E 103°41′57″	*C. nymphaeae*
LMT14	Leaf	Sharpblue	Caichang, Dayi	N 30°30′8″ E 103°41′57″	*C. fructicola*
LMT15	Leaf	Misty	Taiping, Shuangliu	N 30°25′53″ E 104°11′54″	*C. siamense*
LMT16	Leaf	O’Neal	Taiping, Shuangliu	N 30°25′53″ E 104°11′54″	*C. fructicola*
LMT17	Leaf	Misty	Tuqiao, Jintang	N 30°31′17″ E 104°50′5″	*C. fructicola*
LMT18	Leaf	Misty	Tuqiao, Jintang	N 30°31′17″ E 104°50′5″	*C. fructicola*
LMT19	Leaf	Sharpblue	Daguang, Dujiangyan	N 30°51′4″ E 103°34′44″	*C. siamense*
LMT20	Leaf	Sharpblue	Taiping, Shuangliu	N 30°25′53″ E 104°11′54″	*C. fructicola*
LMT21	Leaf	O’Neal	Taiping, Shuangliu	N 30°25′53″ E 104°11′54″	*C. fructicola*
LMT22	Stem	Sharpblue	Caichang, Dayi	N 30°30′8″ E 103°41′57″	*C. fructicola*
LMT23	Leaf	Sharpblue	Caichang, Dayi	N 30°30′8″ E 103°41′57″	*C. fructicola*
LMT24	Leaf	Sharpblue	Caichang, Dayi	N 30°30′8″ E 103°41′57″	*C. siamense*
LMT25	Stem	O’Neal	Wuyang, Pengshan	N 30°11′44″ E 103°52′8″	*C. karstii*
LMT26	Stem	O’Neal	Wuyang, Pengshan	N 30°11′44″ E 103°52′8″	*C. fructicola*
LMT27	Stem	O’Neal	Wuyang, Pengshan	N 30°11′44″ E 103°52′8″	*C. fructicola*
LMT28	Leaf	O’Neal	Taiping, Shuangliu	N 30°25′53″ E 104°11′54″	*C. fructicola*
LMT29	Leaf	O’Neal	Taiping, Shuangliu	N 30°25′53″ E 104°11′54″	*C. fructicola*
LMT30	Leaf	Sharpblue	Caichang, Dayi	N 30°30′8″ E 103°41′57″	*C. fructicola*
LMT31	Leaf	Sharpblue	Caichang, Dayi	N 30°30′8″ E 103°41′57″	*C. fructicola*
LMT32	Leaf	Sharpblue	Caichang, Dayi	N 30°30′8″ E 103°41′57″	*C. fructicola*
LMT33	Leaf	Sharpblue	Caichang, Dayi	N 30°30′8″ E 103°41′57″	*C. siamense*
LMT34	Leaf	O’Neal	Taiping, Shuangliu	N 30°25′53″ E 104°11′54″	*C. siamense*
LMT35	Stem	O’Neal	Gongping, Wenjiang	N 30°43′22″ E 103°49′28″	*C. siamense*
LMT36	Stem	O’Neal	Gongping, Wenjiang	N 30°43′22″ E 103°49′28″	*C. siamense*
LMT37	Stem	O’Neal	Gongping, Wenjiang	N 30°43′22″ E 103°49′28″	*C. fructicola*
LMT38	Stem	O’Neal	Gongping, Wenjiang	N 30°43′22″ E 103°49′28″	*C. fructicola*
LMT39	Stem	O’Neal	Gongping, Wenjiang	N 30°43′22″ E 103°49′28″	*C. fructicola*
LMT40	Stem	O’Neal	Gongping, Wenjiang	N 30°43′22″ E 103°49′28″	*C. fructicola*
LMT41	Stem	O’Neal	Gongping, Wenjiang	N 30°43′22″ E 103°49′28″	*C. fructicola*
LMT42	Stem	Misty	Tuqiao, Jintang	N 30°31′17″ E 104°50′5″	*C. fructicola*
LMT43	Stem	Misty	Tuqiao, Jintang	N 30°31′17″ E 104°50′5″	*C. fructicola*
LMT44	Leaf	O’Neal	Taiping, Shuangliu	N 30°25′53″ E 104°11′54″	*C. siamense*
LMT45	Leaf	O’Neal	Taiping, Shuangliu	N 30°25′53″ E 104°11′54″	*C. fructicola*
LMT46	Leaf	Sharpblue	Daguang, Dujiangyan	N 30°51′4″ E 103°34′44″	*C. fructicola*
LMT47	Leaf	Sharpblue	Daguang, Dujiangyan	N 30°51′4″ E 103°34′44″	*C. fructicola*
LMT48	Stem	O’Neal	Caichang, Dayi	N 30°30′8″ E 103°41′57″	*C. fructicola*
LMT49	Stem	O’Neal	Caichang, Dayi	N 30°30′8″ E 103°41′57″	*C. fructicola*
LMT50	Leaf	Misty	Tuqiao, Jintang	N 30°31′17″ E 104°50′5″	*C. siamense*
LMT51	Leaf	Sharpblue	Daguang, Dujiangyan	N 30°51′4″ E 103°34′44″	*C. sichuaninese*
LMT52	Leaf	Sharpblue	Caichang, Dayi	N 30°30′8″ E 103°41′57″	*C. fructicola*
LMT53	Leaf	Sharpblue	Caichang, Dayi	N 30°30′8″ E 103°41′57″	*C. fructicola*
LMT54	Leaf	O’Neal	Taiping, Shuangliu	N 30°25′53″ E 104°11′54″	*C. fructicola*
LMT55	Leaf	O’Neal	Taiping, Shuangliu	N 30°25′53″ E 104°11′54″	*C. fructicola*
LMT56	Leaf	Misty	Tuqiao, Jintang	N 30°31′17″ E 104°50′5″	*C. siamense*
LMT57	Leaf	Misty	Tuqiao, Jintang	N 30°31′17″ E 104°50′5″	*C. fructicola*
LMT58	Leaf	Sharpblue	Daguang, Dujiangyan	N 30°51′4″ E 103°34′44″	*C. siamense*
LMT59	Leaf	Misty	Taiping, Shuangliu	N 30°25′53″ E 104°11′54″	*C. fructicola*
LMT60	Leaf	Misty	Taiping, Shuangliu	N 30°25′53″ E 104°11′54″	*C. fructicola*
LMT61	Stem	O’Neal	Caichang, Dayi	N 30°30′8″ E 103°41′57″	*C. nymphaeae*
LMT62	Stem	Sharpblue	Wuyang, Pengshan	N 30°11′44″ E 103°52′8″	*C. fructicola*
LMT63	Stem	Sharpblue	Wuyang, Pengshan	N 30°11′44″ E 103°52′8″	*C. siamense*
LMT64	Stem	Sharpblue	Wuyang, Pengshan	N 30°11′44″ E 103°52′8″	*C. fructicola*
LMT65	Leaf	Misty	Taiping, Shuangliu	N 30°25′53″ E 104°11′54″	*C. karstii*
LMT66	Leaf	Misty	Taiping, Shuangliu	N 30°25′53″ E 104°11′54″	*C. siamense*
LMT67	Leaf	O’Neal	Caichang, Dayi	N 30°30′8″ E 103°41′57″	*C. fructicola*
LMT68	Leaf	O’Neal	Caichang, Dayi	N 30°30′8″ E 103°41′57″	*C. fructicola*
LMT69	Leaf	O’Neal	Caichang, Dayi	N 30°30′8″ E 103°41′57″	*C. fructicola*
LMT70	Leaf	O’Neal	Caichang, Dayi	N 30°30′8″ E 103°41′57″	*C. karstii*
LMT71	Leaf	Misty	Taiping, Shuangliu	N 30°25′53″ E 104°11′54″	*C. kahawae*
LMT72	Stem	Misty	Gongping, Wenjiang	N 30°43′22″ E 103°49′28″	*C. fructicola*
LMT73	Stem	Misty	Gongping, Wenjiang	N 30°43′22″ E 103°49′28″	*C. fructicola*
LMT74	Stem	Misty	Gongping, Wenjiang	N 30°43′22″ E 103°49′28″	*C. kahawae*

**Table 2:** Morphological characteristics of *Colletotrichum* species from blueberry in Sichuan, China.

Group	Species	Colony	Growth Rate (mm/d)	Conidia			Appressoria			Mycelial Appressoria
				Length (μm)	Width (μm)	Shape	Length (μm)	Width (μm)	Shape and Color	
1	*Colletotrichum fructicola*	black and gray, reverse black	6.0 ± 0.04 a	13.3 ± 0.21 b	5.4 ± 0.24 a	cylindrical	6.7 ± 0.16 a	5.0 ± 0.17 a	brown to dark black, ovoid to slightly irregular	oval to irregular, light brown to dark brown, margin lobed or deeply lobed
2	*Colletotrichum siamense*	white, reverse pale yellowish	4.3 ± 0.12 c	11.7 ± 0.41 c	5.4 ± 0.21 a	cylindrical	5.7 ± 0.39 b	4.7 ± 0.22 b	brown to dark black, irregular with a crenate edge	irregularly shaped and brown, with a deep, marginal, lobed edge
3	*Colletotrichum kahawae*	pale gray, reverse pale gray	3.4 ± 0.09 d	14.4 ± 0.60 a	5.0 ± 0.94 b	cylindrical	6.7 ± 0.40 a	5.4 ± 0.17 c	brown to dark black, round	ovoid or irregularly shaped, light brown, with a deep, marginal, lobed, or smooth edge
4	*Colletotrichum nymphaeae*	gray, reverse dark gray	6.2 ± 0.08 a	10.5 ± 0.08 d	4.6 ± 0.43 c	fusiform	7.4 ± 0.33 c	5.9 ± 011 d	gray, round	oval, light brown, smooth edge
5	*Colletotrichum sichuaninese*	gray, reverse pale gray	5.5 ± 0.39 b	14.5 ± 0.32 a	5.2 ± 0.30 ab	long cylindrical	12.0 ± 0.56 d	8.8 ± 0.21 e	brown to dark black, ovoid to slightly irregular	oval or round, brown to dark brown, with a smooth border
6	*Colletotrichum karstii*	white, reverse white	6.4 ± 0.05 a	15.7 ± 0.12 e	7.0 ± 0.26 d	fusiform	8.9 ± 0.32 e	7.4 ± 0.46 f	brown to dark black, round	oval to round, light brown, and smooth edges

Note: In a column, averages followed by different small letters indicate statistically significant differences (*p* < 0.05); a–f: the values with the same letter in a column do not significantly differ according to Duncan’s multiple range test.

**Table 3:** Aggressiveness differentiation of *Colletotrichum* species.

Species	Average Lesion Diameter (mm)	
	Detached Stems	Leaves on Seedings
*C. fructicola*	14.2 ± 0.35 a	15.0 ± 0.21 a
*C. siamense*	12.1 ± 0.30 b	2.9 ± 0.21 c
*C. kahawae*	6.2 ± 0.15 e	2.8 ± 0.15 c
*C. nymphaeae*	7.0 ± 0.22 d	5.1 ± 0.21 b
*C. sichuaninese*	6.0 ± 0.22 e	1.6 ± 0.10 d
*C. karstii*	10.1 ± 0.26 c	1.6 ± 0.20 d

Note: In a column, averages followed by different small letters indicate statistically significant differences (*p* < 0.05). a–e: the values with the same letter in a column do not significantly differ according to Duncan’s multiple range test.

## Supplementary Materials

The following are available online at https://www.mdpi.com/2076-0817/9/9/718/s1, Table S1. GenBank accession numbers and sources of different strains used in the current study. Figure S1. A maximum parsimony tree based on partial GAPDH gene sequences from 74 *Colletotrichum* isolates. Parsimony bootstrap values of more than 50% are shown at the nodes. Isolates selected for subsequent phylogenetic analyses are highlighted in red. Figure S2. A maximum likelihood tree based on partial GAPDH gene sequences from 74 *Colletotrichum* isolates. Parsimony bootstrap values of more than 50% are shown at the nodes. Isolates selected for subsequent phylogenetic analyses are highlighted in blue. Figure S3. Phylogenetic tree generated from maximum likelihood analysis based on GAPDH, ITS, TUB2, ACT and CAL gene sequences. Parsimony bootstrap values of more than 50% are shown at the nodes. Isolates from this study are shown in bold, and the species of the same color belong to the same complex species of *Colletotrichum*. The tree is rooted with *Monilochaetes infuscans*. Detailed information is provided in Table S1.
