# Supplementary material for: Identification of Colletotrichum Species Associated with Blueberry Anthracnose in Sichuan, China
Source: Pathogens. 2020 Aug 31;9(9):718. doi: 10.3390/pathogens9090718 (PMC7559709; doi:10.3390/pathogens9090718)
Supplement: Supplementary file 1 [file pathogens-09-00718-s001.pdf]

Supplementary Table S1. GenBank accession numbers and source of different strains used in the current study

species	Strain no.	Host	Location	GenBank accession numbers				
				ITS	TUB2	ACT	GAPDH	CAL
C. acutatum	BRIP28519	*Carica papaya*	Australia	FJ 972601	FJ 907443	FJ 907428	FJ 972580	FJ 917510
C. acutatum	CBS29467	*Carica papaya*	Australia	FJ 972610	FJ 907444	FJ 907429	FJ 972581	FJ 917511
C. boninense	CBS128547*	*Camellia* sp.	New Zealand	JQ005159	JQ005593	JQ005507	JQ005246	JQ005680
C. boninense	CBS123755*	*Crinum asiaticum*	Japan	JQ005153	JQ005588	JQ005501	JQ005240	JQ005674
C. cliviae	CBS 125375	*Clivia miniata*	China	JX519223	JX519249	JX519240	JX546611	-
C. cliviae	CSSK4*	*Clivia miniata*	China	GQ485607	GQ849440	GQ856777	GQ856756	GQ849464
C. cliviae	CSSS1	*Clivia miniata*	China	GU109479	GU085869	GU085861	GU085868	GU085864
C. sichuaninese	LJTJ3	*Capsicum* sp.	China	KP748193	KP823850	KP823738	KP823773	KP823808
C.sichuaninese	LJTJ16	*Capsicum* sp.	China	KP748207	KP823851	KP823739	KP823786	KP823809
C.sichuaninese	LJTJ22	*Capsicum* sp.	China	KP748213	KP823852	KP823740	KP823792	KP823810
C.sichuaninese	LJTJ30	*Capsicum* sp.	China	KP748221	KP823853	KP823741	KP823800	KP823811
C.sichuaninese	**LMT1**	***Blueberry***	**China**	**MF543100**	**MF471719**	**MF471703**	**MF543063**	**MF471739**
C. coccodes	CBS164.49*	*Solanum tuberosum*	Netherlands	HM171678	-	HM171666	HM171672	HM171669
C. coccodes	CBS369.75	*Solanum tuberosum*	Netherlands	HM171679	-	HM171667	HM171673	HM171670
C. coccodes	CPOS1	*Solanum tuberosum*	China	GQ485588	GQ849444	GQ856787	GQ856744	HM171670
C.gloeosporioides	CBS 95397	*Citrus sinensis*	Italy	FJ972609	FJ907445	FJ 907430	FJ 972582	FJ 917512
C.gloeosporioides	CBS953.97*	*Citrus sinensis*	Italy	GQ485605	GQ849434	GQ856782	GQ856762	GQ849452
C.gloeosporioides	IMI356878*	*Citrus sinensis*	Italy	JX010152	JX010445	JX009531	JX010056	JX009731
C.gloeosporioides	CORCG5	*Vanda* sp.	China	HM034809	HM034811	HM034801	HM034807	HM034803

C.gloeosporioides	LJTJ13	*Capsicum sp.*	China	KP748204	KP823863	KP823751	KP823783	KP823821
C. scovillei	LJTJ35	*Capsicum* sp.	China	KP748226	KP823849	KP823735	KP823805	KP823807
C. scovillei	LJTJ42	*Capsicum* sp.	China	KP943572	KP943588	KP943562	KP943516	KP943582
C. scovillei	LJTJ61	*Capsicum* sp.	China	KP943573	KP943589	KP943563	KP943517	KP943583
Monilochaetes infuscans	CBS869.96	*Unknown*	Unknown	JQ005780	JQ005864	JQ005843	-	-
C. karstii	CORCG6*	*Vanda* sp.	China	HM585409	HM585428	HM581995	HM585391	HM582013
C. karstii	CORCK1	*Calanthe*	China	HM585406	HM585424	HM581991	HM585387	HM582010
C. karstii	GM44L01	*Annona muricata*	Colombia	KC512141	KC512204	KC512183	KC506413	KC512225
C. karstii	**LMT25**	***Blueberry***	**China**	**MF543103**	**MF471722**	**MF471706**	**MF543066**	-
C. karstii	**LMT7**	***Blueberry***	**China**	**MF543102**	**MF471721**	**MF471705**	**MF543065**	**MF471741**
C. nymphaeae	CLOK3	*Eriobotrya japonica*	China	KJ534689	KJ534680	KJ534737	KJ534755	KJ534725
C. nymphaeae	CBS115408*	-	-	JQ948212.1	JQ949863.1	JQ949533.1	JQ948542.1	-
C. nymphaeae	CBS515.78	*Nymphaea alba*	Netherlands	JQ948197	JQ949848	JQ949518	JQ948527	-
C. nymphaeae	**LMT13**	***Blueberry***	**China**	**MF543101**	**MF471720**	**MF471704**	**MF543064**	**MF471740**
C. brevisporum	LC0600*	*Neoregalia* sp.	Thailand	JN050238	JN050244	JN050216	JN050227	JN050222
C. brevisporum	LC0870	*Pandanus pygmaeus*	Thailand	JN050239	JN050245	JN050217	JN050228	-
C. brevisporum	LJTJ24	*Capsicum* sp.	China	KP748215	-	KP823736	KP823794	-
C. siamense	LJTJ5	*Capsicum* sp.	China	KP748195	KP823868	KP823756	KP823775	KP823825
C. siamense	LJTJ7	*Capsicum* sp.	China	KP748198	KP823869	KP823757	KP823777	KP823826
C. siamense	**LMT15**	***Blueberry***	**China**	**MF543104**	**MF471723**	**MF471707**	**MF543067**	-
C. siamense	**LMT24**	***Blueberry***	**China**	**MF543106**	**MF471724**	**MF471708**	**MF543068**	**MF471742**
C. siamense	**LMT33**	***Blueberry***	**China**	**MF543107**	**MF471725**	**MF471709**	**MF543069**	**MF471743**
C. siamense	**LMT35**	***Blueberry***	**China**	**MF543108**	**MF471726**	**MN311515**	**MF543072**	**MF471744**
C. siamense	C1315.2*	*coffea arabica*	Thailand	JX010171	JX010404	JX009518	JX009924	-

C. siamense	C1263.11*	*Persea americana*	South Africa	JX010162	JX010393	JX009506	JX010051	JX009703
C. siamense	**LMT34**	***Blueberry***	**China**	**MN295188**	**MN295203**	**MN311500**	**MF543070**	-
C. siamense	**LMT19**	***Blueberry***	**China**	**MF543105**	**MN295202**	-	**MF543071**	-
C. kahawae	C1275.1*	*coffee arabica*	Angola	JX010234	JX010435	JX009474	JX010040	JX009638
C. kahawae	Q025*	*Blackberry*	Colombia	KJ001121	KJ001125	KJ001103	KJ001114	KJ001107
C. kahawae	C1252.12	*Kunzea ericoides*	New Zealand	JX010227	JX010427	JX009473	JX009904	-
C. kahawae	**LMT4**	***Blueberry***	**China**	**MF543109**	**MF471727**	**MF471710**	**MF543073**	**MF471745**
C. kahawae	**LMT11**	***Blueberry***	**China**	**MF543110**	**MF471728**	**MF471711**	**MF543074**	**MF471746**
C. fructicola	**LMT3**	***Blueberry***	**China**	**MF543111**	**MF471729**	**MF471712**	**MF543075**	**MF471747**
C. fructicola	**LMT5**	***Blueberry***	**China**	**MF543112**	**MF471730**	**MF471713**	**MF543076**	**MF471748**
C. fructicola	**LMT6**	***Blueberry***	**China**	**MF543113**	**MF471731**	**MF471714**	**MF543077**	**MF471749**
C. fructicola	**LMT9**	***Blueberry***	**China**	**MF543114**	**MF471732**	**MF471715**	**MF543079**	**MF471750**
C. fructicola	**LMT12**	***Blueberry***	**China**	**MF543115**	**MF471733**	**MF471716**	**MF543081**	**MF471751**
C. fructicola	**LMT14**	***Blueberry***	**China**	**MF543116**	**MF471734**	**MF471717**	**MF543082**	**MF471752**
C. fructicola	**LMT31**	***Blueberry***	**China**	**MF543117**	**MF471735**	**MF471718**	**MF543094**	-
C. fructicola	**LMT36**	***Blueberry***	**China**	**MF543118**	**MF471736**	**MN311516**	**MF543096**	**MF471753**
C. fructicola	**LMT38**	***Blueberry***	**China**	**MF543119**	**MF471737**	**MN311517**	**MF543097**	**MF471754**
C. fructicola	**LMT39**	***Blueberry***	**China**	**MF543120**	**MF471738**	**MN311518**	**MF543098**	**MF471755**
C. fructicola	**LMT41**	***Blueberry***	**China**	**MF543121**	**MN295218**	**MN311514**	**MF543099**	**MF471756**
C. fructicola	**LMT8**	***Blueberry***	**China**	**MN295197**	**MN295212**	**MN311509**	**MF543078**	-
C. fructicola	**LMT10**	***Blueberry***	**China**	**MN295192**	**MN295207**	**MN311504**	**MF543080**	**MN328264**
C. fructicola	**LMT16**	***Blueberry***	**China**	**MN295198**	**MN295213**	**MN311510**	**MF543083**	-
C. fructicola	**LMT17**	***Blueberry***	**China**	**MF543113**	**MN295214**	-	**MF543084**	-
C. fructicola	**LMT18**	***Blueberry***	**China**	**MN295195**	**MN295210**	**MN311507**	**MF543085**	-

C. fructicola	**LMT20**	***Blueberry***	**China**	**MN295191**	**MN295206**	**MN311503**	**MF543086**	**MN328263**
C. fructicola	**LMT21**	***Blueberry***	**China**	**MN295189**	**MN295204**	**MN311501**	**MF543087**	-
C. fructicola	**LMT22**	***Blueberry***	**China**	**MN295196**	**MN295211**	**MN311508**	**MF543088**	-
C. fructicola	**LMT26**	***Blueberry***	**China**	**MN295190**	**MN295205**	**MN311502**	**MF543089**	**MN328262**
C. fructicola	**LMT27**	***Blueberry***	**China**	**MN295193**	**MN295208**	**MN311505**	**MF543090**	**MN328265**
C. fructicola	**LMT28**	***Blueberry***	**China**	**MN295201**	**MN295217**	**MN311513**	**MF543091**	-
C. fructicola	**LMT29**	***Blueberry***	**China**	**MN295194**	**MN295209**	**MN311506**	**MF543092**	**MN328266**
C. fructicola	**LMT30**	***Blueberry***	**China**	**MN295199**	**MN295215**	**MN311511**	**MF543093**	-
C. fructicola	**LMT32**	***Blueberry***	**China**	**MN295200**	**MN295216**	**MN311512**	**MF543095**	-
C. fructicola	C1275.7	*Ficus edulis*	Gemany	JX010181	JX010400	JX009495	JX009923	JX009671
C. fructicola	C1316.21	*Tetragastris*	Panama	JX010173	JX010409	JX009581	JX010032	JX009674
C. fructicola	FAFU01	*Jute*	China	KT439373	KT439339	KT439346	KT439359	KT439352
C. fructicola	BPDI16*	*coffea arabica*	Thailand	FJ972603	FJ907441	FJ907426	FJ972578	-
C. fructicola	LJTJ2	*Capsicum* sp.	China	KP748192	KP823854	KP823742	KP823772	KP823812
C.chrysophilum	CMM4268*	*Musa sp.*	Brazil	KX094252	KX094285	KX093982	KX094183	KX094063
C.chrysophilum	CMM4292	*Musa sp.*	Brazil	KX094248	KX094284	KX093981	KX094182	KX094062
C.chrysophilum	CMM4387	*Musa sp.*	Brazil	KX094246	KX094281	KX093980	KX094181	KX094061
C.chrysophilum	CMM4394	*Musa sp.*	Brazil	KX094239	KX094282	KX093979	KX094179	KX094059
C.noveboracense	AFKH109*	*Apple*	Columbia	MN646685	MN640569	MN640565	MN640567	MN640566
C.noveboracense	AFK220	*Apple*	Ulster	MN625451	MN622861	MN622839	MN689180	MN622851
C.noveboracense	AFK423	*Apple*	Ulster	MN708219	MN701194	MN701181	MN741085	MN701191
C.fioriniae	CBS128517*	-	USA	MH865005	JQ949943	JQ949613	JQ948622	-
C.nupharicola	CBS470.96*	*Nuphar lutea subsp.*	USA	JX010187	JX010398	JX009437	JX009972	JX009663
C.nupharicola	CBS472.96	*Nymphaea ordorata*	USA	JX010188	JX010399	JX009582	JX010031	JX009662

C.horii	ICMP10492*	*Diospyros kaki*	Japan	-	JQ071915	JQ071911	JX009964	JX009604
C.horii	ICMP17970	*Diospyros kaki*	Japan	JX010213	-	JX009467	-	-
C.horii	ICMP17968	*Diospyros kaki*	China	JX010212	JX010378	JX009547	-	JX009606
C.tropicale	ICMP18653*	*Theobroma cacao*	Panama	JX010264	JX010407	JX009489	JX010007	JX009719
C.tropicale	ICMP18672	*Litchi chinensis*	Japan	JX010275	JX010396	JX009480	JX010020	JX009722
C.tropicale	ICMP18651	*Annona muricata*	Panama	JX010277	-	JX009570	JX010014	JX009720
C.asianum	ICMP18580*	*Coffea arabica*	Thailand	FJ972612	JX010406	JX009584	JX010053	FJ917506
C.asianum	ICMP18603	*Mangifera indica*	Philippines	JX010195	-	JX009579	JX009938	JX009725
C.asianum	ICMP18605	*Mangifera indica*	Thailand	JX010194	-	JX009465	JX010021	JX009726
C.musae	ICMP19119*	*Musa sp*	USA	JX010146	HQ596280	JX009433	JX010050	JX009742
C.musae	ICMP18600	*Musa sp*	Philippines	JX010144	-	JX009556	JX010038	JX009686
C.musae	ICMP12930	*Musa sp*	New Zealand	JX010141	-	JX009566	JX009986	JX009685
C.musae	ICMP18701	*Musa sp*	Philippines	JX010145	-	JX009551	JX010047	JX009687
C.aenigma	ICMP18608*	*Persea americana*	Israel	JX010244	JX010389	JX009443	JX010044	JX009683
C.aenigma	ICMP18686	*Pyrus pyrifolia*	Japan	JX010243	JX010390	JX009519	JX009913	JX009684
C.aenigma	GA415	*Avocado*	Israel	KX620327	KX620360	KX620164	KX620261	KX620226
C.queenslandicum	ICMP1778*	*Carica papaya*	Australia	JX010276	JX010414	JX009447	JX009934	JX009691
C.queenslandicum	ICMP1780	*Carica sp.*	Australia	JX010186	-	JX009504	JX010010	JX009693
C.queenslandicum	ICMP12564	*Persea americana*	Australia	JX010184	-	JX009573	JX009919	JX009692
C. cymbidiicola	MI347923*	*Cymbidium sp.*	Australia	JQ005166	JQ005600	JQ005514	JQ005253	JQ005687
C. cymbidiicola	ICMP18584	*Cymbidium sp.*	New Zealand	JQ005167	JQ005601	JQ005515	JQ005254	JQ005688
C. cymbidiicola	CBS130241	*Dacrycarpus*	New Zealand	JQ005236	JQ005670	JQ005584	JQ005323	JQ005757
C. petchii	CBS378.94*	*Dracaena marginata*	Italy	JQ005223	JQ005657	JQ005571	JQ005310	JQ005744
C. petchii	CBS118193	*Dracaena sanderana*	China	JQ005227	JQ005661	JQ005575	JQ005314	JQ005748

C. petchii	CBS118774	*Dracaena sanderana*	China	JQ005225	JQ005659	JQ005573	JQ005312	JQ005746
C. cuscutae	CPC18873*	*Cuscuta sp.*	Dominica	JQ948195	JQ949846	JQ949516	JQ948525	-
C. godetiae	CBS133.44*	*Clarkia hybrida, cv*	Denmark	JQ948402	JQ950053	JQ949723	JQ948733	-
C. godetiae	CBS160.50	*Citrus aurantium*	-	JQ948406	JQ950057	JQ949727	JQ948737	-
C. godetiae	IMI351248	*Ceanothus sp.*	UK	JQ948433	JQ950084	JQ949754	JQ948764	-
C. viniferum	C4	*Vitis vinifera*	China	KF156848	KF288968	KF377516	KF377479	-
C. viniferum	G141-1	*Vitis vinifera*	China	**KF156847**	KF288967	KF377514	KF377477	-
C. viniferum	B12	*Vitis vinifera*	China	KF156850	KF288969	KF377518	KF377481	-
C. viniferum	G12-1	*Vitis vinifera*	China	KF156846	KF288962	KF377513	KF377476	-

Note: ITS=internal transcribed spacer regions, TUB2= β-tubulin, ACT=actin, GAPDH=glyceraldehyde-3-phosphate dehydrogenase, CAL=calmodulin.

Asterisks (*) indicate ex-holotype or ex-epitype cultures. Isolates from this study are shown in bold.

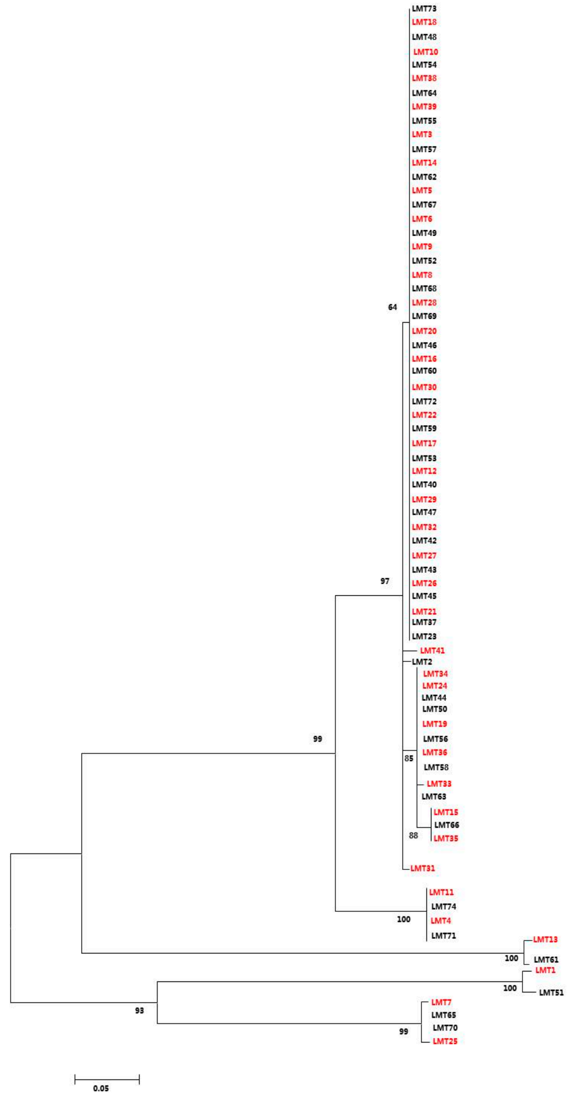

Figure S1. A maximum parsimony tree based on partial GAPDH gene sequences from 74 *Colletotrichum* isolates. Parsimony bootstrap values of more than 50%

are shown at the nodes. Isolates selected for subsequent phylogenetic analyses are highlighted in red.

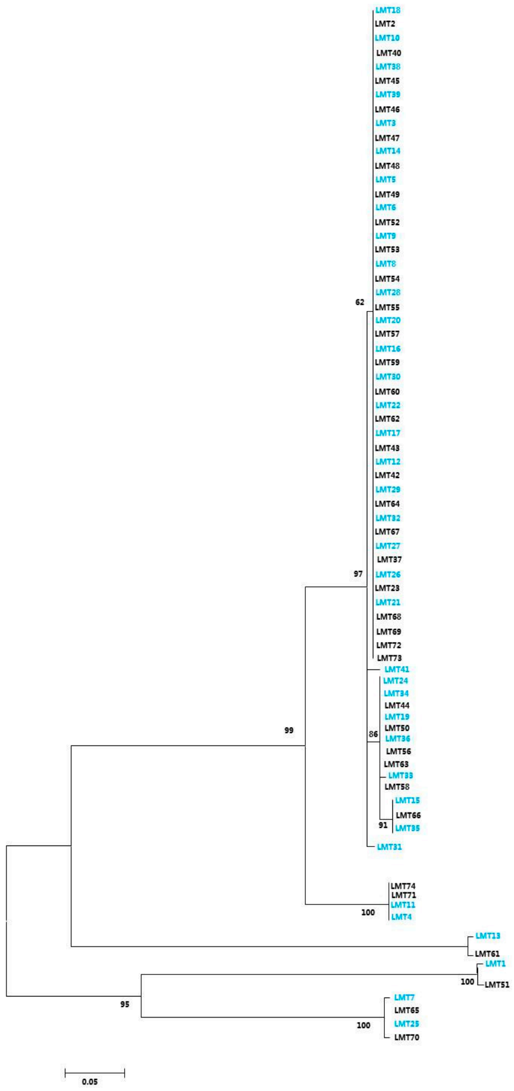

LMT18
LMT2
LMT10
LMT40
LMT38
LMT45
LMT39
LMT46
LMT3
LMT47
LMT14
LMT48
LMT5
LMT49
LMT6
LMT52
LMT9
LMT53
LMT8
LMT54
LMT28
62
LMT55
LMT20
LMT57
LMT16
LMT59
LMT30
LMT60
LMT22
LMT62
LMT17
LMT43
LMT12
LMT42
LMT29
LMT64
LMT32
LMT67
LMT27
LMT37
97
LMT26
LMT23
LMT21
LMT68
LMT69
LMT72
LMT73
LMT41
LMT24
LMT34
LMT44
LMT19
LMT50
99
86
LMT36
LMT56
LMT63
LMT33
LMT58
LMT15
LMT66
91
LMT35
LMT31
LMT74
LMT71
LMT11
100
LMT4
LMT13
LMT61
LMT1
100
LMT51
LMT7
95
LMT65
LMT25
100
LMT70
0.05

Figure S2. A maximum likelihood tree based on partial GAPDH gene sequences from 74 *Colletotrichum* isolates. Parsimony bootstrap values of more than 50% are shown at the nodes. Isolates selected for subsequent phylogenetic analyses are highlighted in blue.

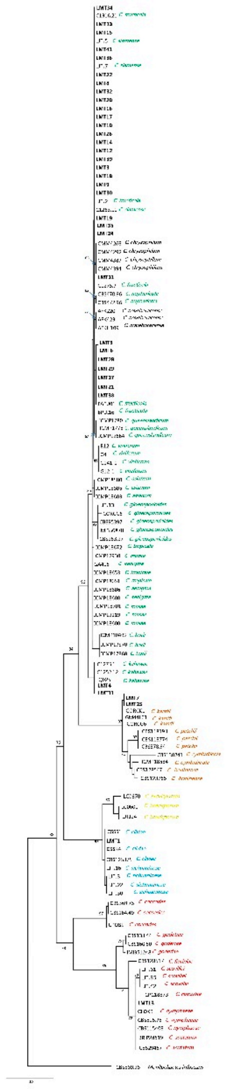

Figure S3. Phylogenetic tree generated from maximum likelihood analysis based on GAPDH, ITS, TUB2, ACT and CAL gene sequences. Parsimony bootstrap values of more than 50% are shown at the nodes. Isolates from this study are shown in bold, and the species of the same color belong to the same complex species of *Colletotrichum*. The tree is rooted with *Monilochaetes infuscans*. Detailed information is provided in Table S1.
